# Computed tomography-based radiomics prediction model for differentiating invasive pulmonary aspergillosis and *Pneumocystis jirovecii* pneumonia

**DOI:** 10.3389/fcimb.2025.1552556

**Published:** 2025-07-10

**Authors:** Zhiguo Peng, Xingzhe Gao, Miao He, Xinyue Dong, Dongdong Wang, Zhengjun Dai, Dexin Yu, Huaibin Sun, Jun Tian, Yu Hu

**Affiliations:** ^1^ Department of Organ Transplantation, Qilu Hospital, Cheeloo College of Medicine, Shandong University, Jinan, China; ^2^ Department of Anesthesiology, Qilu Hospital (Qingdao), Cheeloo College of Medicine, Shandong University, Qingdao, China; ^3^ Department of Medical Oncology, Qilu Hospital (Qingdao), Cheeloo College of Medicine, Shandong University, Qingdao, China; ^4^ Department of Oncology, Qilu Hospital of Shandong University, Dezhou Hospital, Dezhou, China; ^5^ Department of Radiology, Qilu Hospital, Cheeloo College of Medicine, Shandong University, Jinan, China; ^6^ Scientific Research Department, Huiying Medical Technology Co., Ltd, Beijing, China; ^7^ Department of Oncology, Qilu Hospital, Cheeloo College of Medicine, Shandong University, Jinan, China

**Keywords:** invasive pulmonary aspergillosis, *Pneumocystis jirovecii* pneumonia, discriminant model, radiomics, CT

## Abstract

**Background:**

*Pneumocystis jirovecii* and *Aspergillus fumigatus* are important pathogens that cause fungal pulmonary infections. Because the manifestations of *P. jirovecii* pneumonia (PJP) or invasive pulmonary aspergillosis (IPA) are difficult to differentiate on computed tomography (CT) images and the treatment of the two diseases is different, correct imaging for diagnosis is highly significant. The present study developed and validated the diagnostic performance of a CT-based radiomics prediction model for differentiating IPA from PJP.

**Methods:**

In total, 97 patients, 51 with IPA and 46 with PJP, were included in this study. Each patient underwent a non-enhanced chest CT examination. All the patients were randomly divided into two cohorts, training and validation, at a ratio of 7:3 using random seeds automatically generated using the RadCloud platform. Image segmentation, feature extraction, and radiomic feature selection were performed on the RadCloud platform. The regions of interest (ROIs) were manually segmented, including the consolidation area with the surrounding ground-glass opacity (GGO) area and the consolidation area alone. Six supervised-learning classifiers were used to develop a CT-based radiomics prediction model, which was estimated using the receiver operating characteristic (ROC) curve, area under the curve (AUC), sensitivity, specificity, precision, and F1-score. The radiomics score was also calculated to compare the prediction performance.

**Results:**

Classifiers trained with the consolidation area and surrounding GGO area as the ROI showed better prediction efficacy than classifiers trained using only the consolidation area as the ROI. The XGBoost model performed better than the other classifiers and radiomics scores in the validation cohort, with an AUC of 0.808 (95% CI, 0.655–0.961).

**Conclusions:**

This radiomics model can effectively assist in the differential diagnosis of PJP and IPA. The consolidation area with the surrounding GGO area was more suitable for ROI segmentation.

## Introduction

Invasive pulmonary aspergillosis (IPA) is among the most severe disorders and has the highest mortality rate among all types of pulmonary aspergillosis (Dai et al., 2013). IPA characteristically occurs in immunocompromised individuals, including those with long-term neutropenia, hematological malignancies, or solid organ or hematopoietic stem cell transplantation. IPA is difficult to diagnose and treat and can cause symptoms such as coughing, chest pain, hemoptysis, severe breathing difficulties, and even respiratory failure (Schweer et al., 2016).

*Pneumocystis jirovecii* is a specific fungal pathogen that can cause *P. jirovecii* pneumonia (PJP), a severe opportunistic pneumonia, in immunocompromised patients, including those with organ transplantation, cancer, and malignant hematological diseases (Avino et al., 2016). The typical symptoms of PJP include fever, cough, and hypoxemia, which can rapidly aggravate and lead to severe respiratory failure. Non-enhanced chest computed tomography (CT) is often used to diagnose patients with suspected PJP or IPA.

Typical CT manifestations of IPA include nodules, ground-glass opacities (GGOs), segmental consolidations, pleural effusions, or cavities (Kuhlman et al., 1988). Typical imaging findings of PJP include ground-glass density shadows in the lungs, which are diffusely distributed, scattered, or interlaced with the normal lung tissue. GGOs in the lungs can merge into nodular or massive consolidated opacities. However, the manifestations of PJP and IPA are non-specific and are sometimes difficult to differentiate clinically (Roux et al., 2014). Because the manifestations of PJP or IPA are difficult to differentiate on CT images, and the treatment of the two diseases is quite different, correct imaging for diagnosis may allow for early initiation of treatment before microbiological confirmation and significantly improve prognosis.

In cases where distinguishing between PJP and IP caused by *Aspergillus fumigatus* remains challenging based on galactomannan (GM) assay, β-D-glucan (BDG) testing, and macroscopic imaging findings, a nuanced diagnostic approach is warranted. Both GM antigen (specific to *Aspergillus*) and BDG (elevated in PJP and other fungal infections) exhibit overlapping limitations: BDG lacks species specificity, while GM sensitivity may decline in non-neutropenic hosts or with prophylactic antifungal use. Radiologically, atypical presentations, such as diffuse ground-glass opacities mimicking PJP in early IPA or focal consolidation overlapping with bacterial pneumonia, further obscure differentiation.

To resolve this diagnostic ambiguity, integrating host immune status with ancillary biomarkers is critical. For example, profound CD4+ lymphopenia (<200/μL) strongly favors PJP in patients with HIV, whereas prolonged neutropenia or hematopoietic stem cell transplantation heightens IPA probability. Molecular assays [e.g., *P. jirovecii* PCR on bronchoalveolar lavage fluid (BALF) or *Aspergillus*-specific PCR] enhance pathogen detection when conventional methods are inconclusive. Histopathological confirmation via biopsy, demonstrating *Pneumocystis* cysts (methenamine silver stain) or septate hyphae with acute-angle branching (Grocott’s stain), remains the diagnostic gold standard but is often limited by procedural risks.

Ultimately, a tiered diagnostic algorithm combining risk stratification, serial biomarker monitoring, and advanced imaging techniques (e.g., dynamic contrast-enhanced CT for IPA) is essential to mitigate misdiagnosis and guide timely, species-directed therapy.

Radiomics is an emerging imaging technology designed to convert clinical digital images into high-dimensional, mineable data via high-throughput extraction of the quantitative features (Gillies et al., 2016). This technique has been applied to predict pathological classification and differential diagnosis or to assess gene expression, response to therapy, and disease prognosis (Limkin et al., 2017).

To the best of our knowledge, this study is the first to investigate the role of radiomics in improving early differential diagnosis between IPA and PJP. The purpose of our study was to investigate the feasibility of using a CT-based radiomics model to differentiate IPA from PJP.

## Materials and methods

### Patient population

Patient information and data from the Picture Archiving and Communication System (PACS) in Qilu Hospital of Shandong University from September 2013 to May 2022 were collected. Patients with a positive metagenomic next-generation sequencing (mNGS) result obtained using BALF as the detection sample and a non-enhanced thorax CT scan were included. The inclusion criteria were as follows: (1) *P. jirovecii* or *Aspergillus* infection was confirmed using mNGS, and the corresponding treatment was effective; (2) Regular dosage of non-enhanced chest CT revealed that the lesions were mainly consolidations and GGOs. The exclusion criteria were as follows: (1) The imaging diagnosis and evaluation were consistent with the mNGS analysis reports; (2) Motion artifacts in CT images, poor image quality, and large differences in scanning conditions; (3) The pathogen was not confirmed due to ineffective treatment or clinical suspicion of a mixed infection; (4) Other pneumonia, illness, or unconfirmed pneumonia. Finally, 97 patients, including 46 in the PJP group and 51 in the IPA group, were included in this retrospective study. Prior information on sample size can be obtained from previous studies. A review analyzing the sample size of some studies using radiomics showed that in a total of 87 studies that were included in the final report, most had sample sizes above 50 patients, with a median cohort size of 101 (Dercle et al., 2022). Therefore, 97 patients were deemed adequate for this study. Data on their clinical characteristics, including sex and age at diagnosis, were also collected. According to a classic review, the ratio used in practice ranges from 60:40 to 90:10 (Shur et al., 2021). As in many other studies, a one-third proportion represents a trade-off between having sufficient data in the training set to ensure that the model has sufficient predictive power and a sufficiently large test dataset to ensure that the predicted performance estimate is accurate (Chen et al., 2022; Jiang et al., 2022; Zheng et al., 2022). All the patients were randomly divided into training and validation cohorts at a ratio of 7:3 using random seeds automatically generated by the RadCloud platform. No differences were observed between the training and validation sets.

This study was approved by the Medical Ethics Committee of Qilu Hospital of Shandong University (KYLL-202209-016).

### Image acquisition

All thoracic CT images were obtained with a Siemens SOMATOM Definition AS 64-slice spiral CT, scanning from the thoracic inlet to the diaphragm, with the following parameter settings: tube voltage 120KV, tube current 250–400mA/s (automatic tube current modulation was used); 18–35cm field of view; 512×512 reconstruction matrix; 5mm slice thickness, 1.0s scanning time.

### Image segmentation and feature extraction

**Figure 1:**
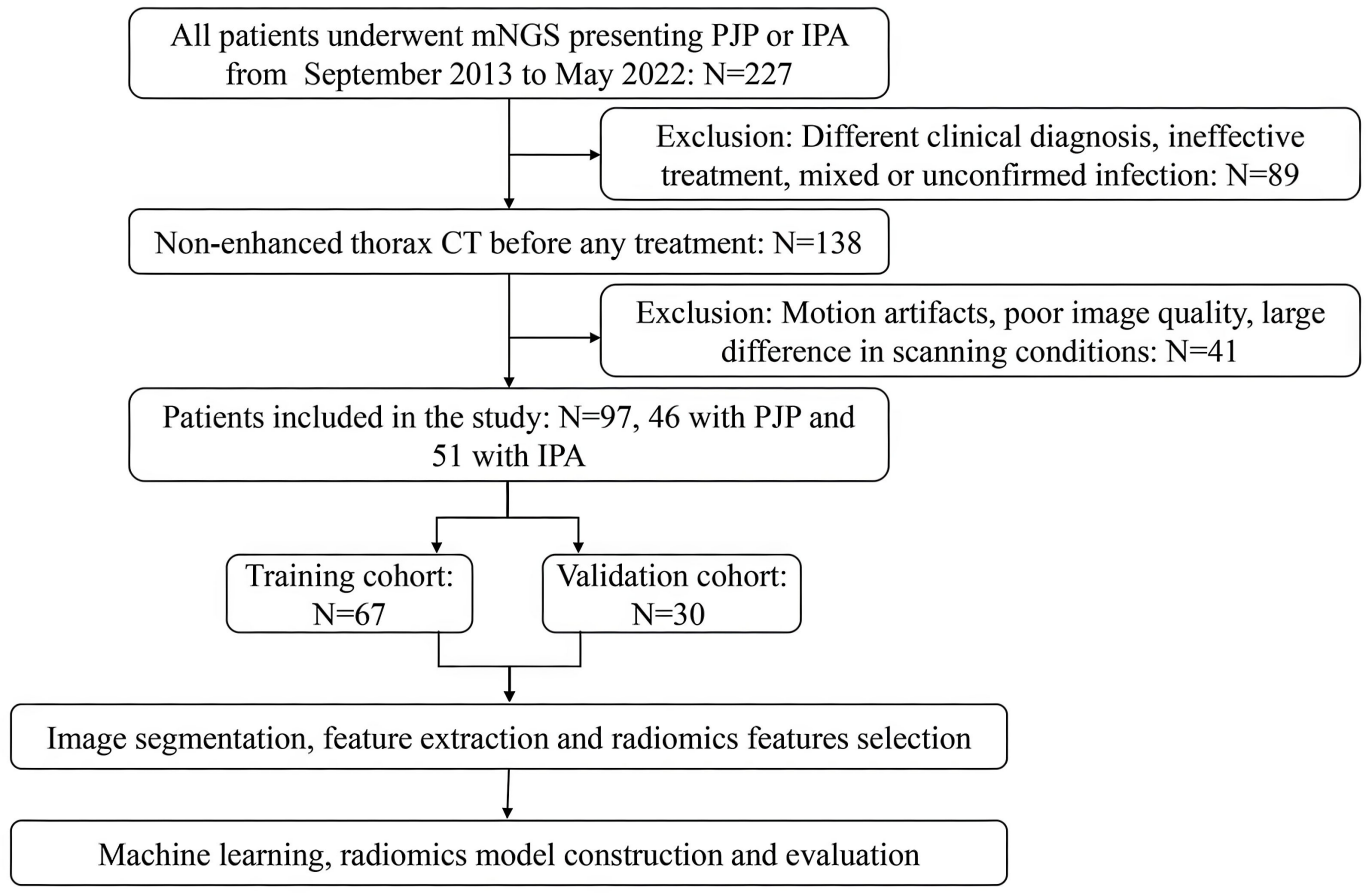
Flowchart of patient recruitment and radiomics model construction.

A flowchart of patient recruitment and radiomics is shown in **Figure 1**. All CT images were saved in the Digital Imaging and Communications in Medicine (DICOM) format and were uploaded to the RadCloud (Version 7.2, Huiying Medical Technology Beijing Co., Ltd) (Yayuan et al., 2021) platform (https://mics.huiyihuiying.com/login?redirect=%2F, Huiying Medical Technology Co., Ltd). RadCloud’s module is based on the pseudo-random number algorithm from the Python language’s random library. By fixing the seed, it can ensure the division of the training and testing sets, thereby ensuring the reproducibility of experimental results. The random seed value used in this research was 803. It uses cloud computing, big data analysis, and machine learning algorithms to manage the DICOM imaging data on cloud platforms. We employed the RadCloud platform because the convenience of its remote operation. Many high-quality studies have been based on the RadCloud platform (Cangir et al., 2022; Wang et al., 2022; Zhao et al., 2022).

**Figure 2:**
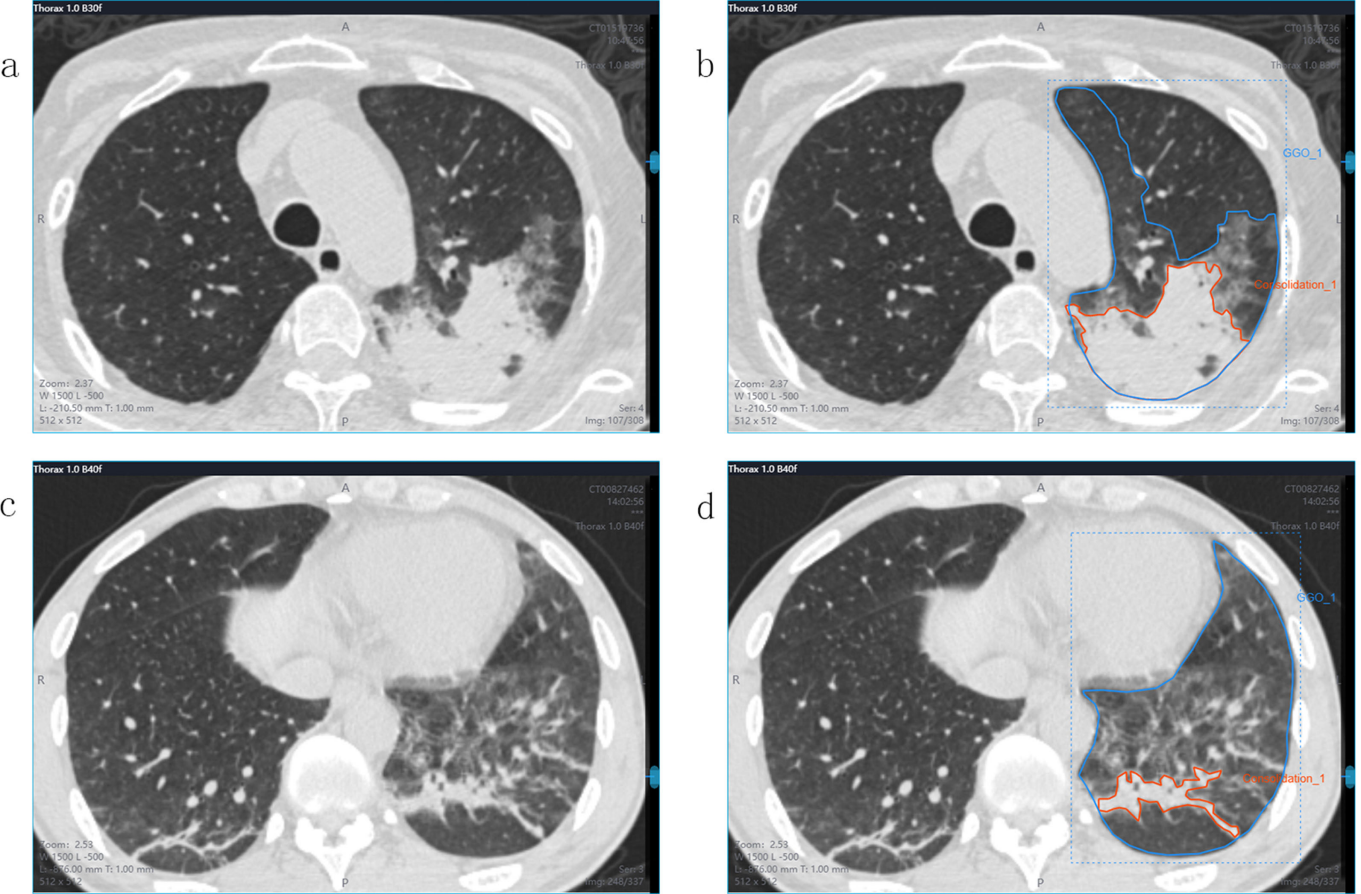
CT images and ROI segmentation. Panels **(a, b)** showed a 55-year-old male with IPA, while panels **(c, d)** showed a 36-year-old male with PJP. Both patients presented similar consolidations and surrounding GGO areas on CT images. panels **(b, d)** show the ROI segmentation method. The orange lines include only the consolidation area as the ROI. The blue lines include both the consolidation area and the GGO area as the ROI.

The regions of interest (ROIs) were manually marked by a radiologist with 5 years of experience and then re-examined by another radiologist with more than 10 years of experience. The radiologists were blinded to the patients’ diagnoses. If there were any contradictions, the senior radiologists evaluated the ROI again to reach an agreement. To better separate the consolidation and surrounding GGO areas from normal lung tissues, we chose the lung window during ROI segmentation. One image that contained the most typical lesion area was selected for each patient and separated as the ROI. Two methods were used to mark the ROIs in each patient. The first method included only the consolidation area. Cavities were included, but the adjacent mediastinum, pleura, and pleural effusion were avoided in the ROIs. In the second method, the consolidation areas with surrounding GGO areas were included. The ROI delineation in the PJP and IPA CT images is presented in **Figure 2**. Finally, 97 ROIs were segmented from the 97 patients. After 3 months, 30 cases were randomly selected, and the two radiologists repeated the segmentation to evaluate the inter- and intra-examiner reliability. Radiomic features were automatically extracted by the platform.

### Radiomic feature selection and radiomics model building

For each CT sequence in each image,1,409 radiomic features were extracted using the RadCloud platform. The obtained features were divided into three categories. First-order statistics consisted of 270 descriptors that quantitatively delineate the distribution of voxel intensities within the CT image through commonly used and basic metrics. The shape- and size-based features contained 14 three-dimensional features that reflected the shape and size of the region. Texture features that can quantify regional heterogeneity differences include 1125 textural features such as the grey-level run-length (GLRLM), grey-level co-occurrence (GLCM), grey-level size zone (GLSZM), neighboring gray-tone difference (NGTDM), and grey-level dependence matrices (GLDM).

The values of the radiomic features were normalized using the z-score method, and stable features with an ICC >0.75 were retained and normalized for subsequent analysis. Dimensionality reduction and the selection of task-specific features were also performed on the RadCloud platform. The feature selection methods included the variance threshold (variance threshold=0.8), SelectKBest, and least absolute shrinkage and selection operator (LASSO) methods. LASSO regression is an improved linear regression method that introduces the L1 regularization penalty term to automatically screen key features and prevent model overfitting while fitting data. The core idea is to optimize a loss function that consists of two parts: one is the sum of squared errors in traditional linear regression (which measures the deviation between predicted values and true values), and the other is the penalty term for the sum of absolute values of all regression coefficients. By adjusting the hyperparameter lambda to control the penalty intensity, as the larger the lambda, the stronger the compression of insignificant features in the model, some coefficients will be directly reset to zero, thus achieving feature selection. This method is particularly suitable for high-dimensional data with a much larger number of features than the sample size (such as gene expression or text analysis), which can improve model interpretability while retaining key variables. In practical applications, the optimal value of λ is usually determined through cross-validation to balance model complexity and prediction accuracy. Based on the variance threshold method, the values of the variance < 0.8 were removed. The SelectKBest method used a p-value to analyze the relationship between the features and classification results, which belong to a single variable feature selection method. Only features with p-values < 0.05 were reserved. The L1 regularizer was used as the cost function in the LASSO model. The error value of the cross-validation was 5, and the maximum number of iterations was 1,000. The radiomics score (radscore) was the sum of the features retained after the LASSO method was multiplied by the corresponding coefficients.

After dimensionality reduction and radiomic features selection, we used six supervised machine learning classifiers for optimal radiomics model construction and selection, including K-nearest neighbors (KNN), support vector machine (SVM), eXtreme Gradient Boosting (XGBoost), random forest (RF), logistic regression (LR), and decision tree (DT). A validation method was used to improve the effectiveness of the model.

### Statistical analysis

The area under the curve (AUC) and receiver operating characteristic (ROC) curve were used to estimate the predictive performance, and the sensitivity, specificity, precision, and F1-score [F1-score = precision × recall × 2/(precision + recall)] were calculated using the platform mentioned above to evaluate the performance of the classifier used in this research. Statistical analyses with clinical characteristics were conducted using SPSS 25.0. The data were expressed as median and interquartile range or mean ± SD. The t-test was applied for normally distributed data, whereas the rank-sum test was used for non-normally distributed data. Statistical significance was set at P < 0.05.

## Results

### Patient characteristics

This study included 97 patients, of whom 51 had IPA and 46 had PJP. There were no significant differences in sex between the IPA and PJP groups. The patients with PJP were significantly younger than those with IPA (**Table 1**).

**Table 1:** Patient characteristics.

Case Profile	*Pneumocystis jirovecii* pneumonia	Invasive pulmonary aspergillosis	Statistics value	P
Number of cases	46	51	–	–
Sex (male/female)	30/16	27/24	χ²=1.504	0.220
Age	53 (37.25)	62 (19)	Z=-3.379	0.001

Values are presented as median and interquartile range.

### Radiomics features and model development

**Figure 3:**
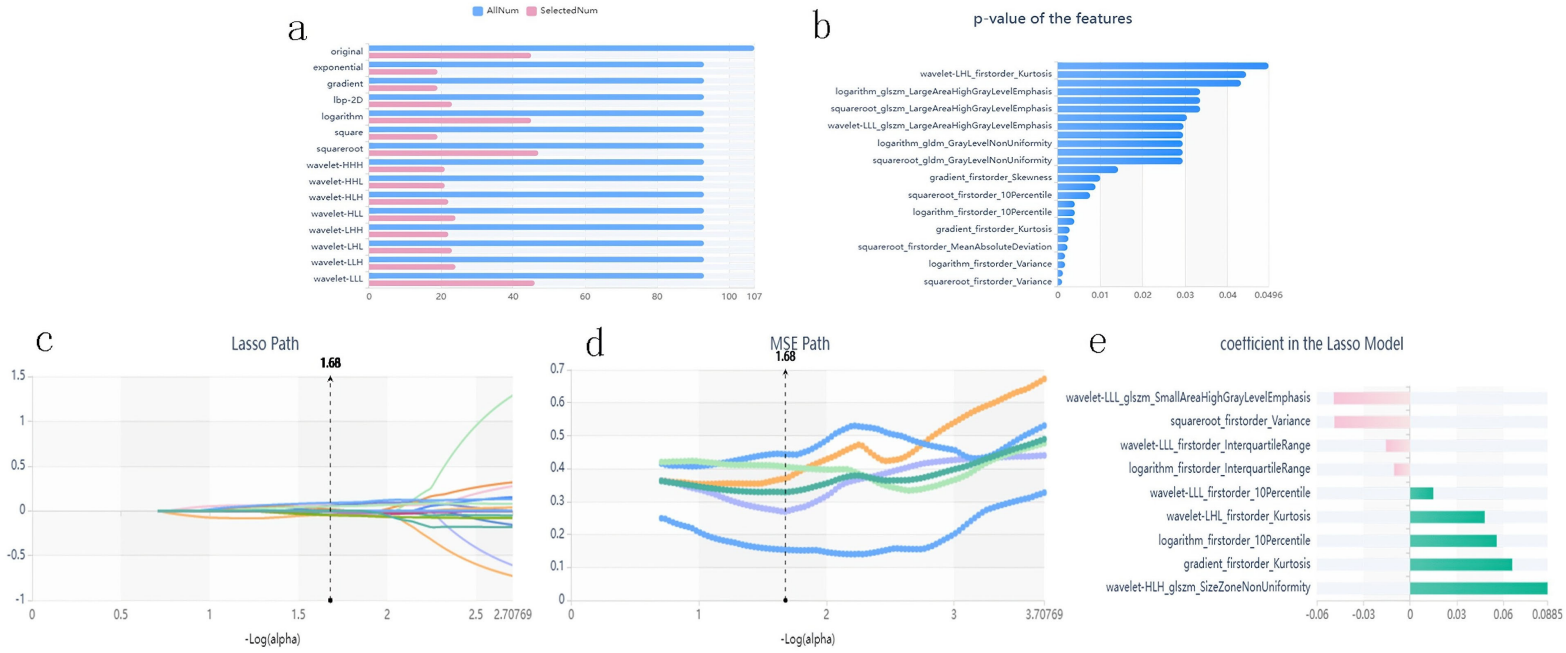
Panel **(a)** shows the variance threshold method on radiomics feature selection. The blue bars show all the radiomics features extracted in CT images, while the pink bars represent the features reserved after variance threshold selection. Panel **(b)** shows the SelectKBest method results for further selecting radiomics features. Only features with p-values smaller than 0.05 were reserved. Panels **(c–e)** are the LASSO path, the Mean Squared Error (MSE) path, and the finally screened features with their coefficients in the LASSO model for feature selection, respectively.

Stable features with an ICC > 0.75 were retained and normalized for subsequent analysis. The AUC of the XGBoost classifier with an ROI that included the consolidation area and surrounding GGO area was 0.808, which was better than that of the classifiers with an ROI only including the consolidation area. Using the variance threshold method, 420 features were selected from 1,409 features (**Figure 3a**), and 26 features were screened out using the selected K best method (**Figure 3b**). Finally, nine optimal features were selected using the LASSO method (**Figures 3c–e**). The radiomic features retained after dimension reduction using the LASSO method are listed in **Table 2**. In the consolidation area with the surrounding GGO area.

**Table 2:** Radiomics features selected with the region of interest (ROI) including the consolidation and ground-glass opacity (GGO) areas.

Radiomics feature	Radiomic class	Filter
Variance	firstorder	squareroot
InterquartileRange	firstorder	logarithm
Kurtosis	firstorder	gradient
10Percentile	firstorder	logarithm
10Percentile	firstorder	wavelet-LLL
InterquartileRange	firstorder	wavelet-LLL
SmallAreaHighGrayLevelEmphasis	glszm	wavelet-LLL
SizeZoneNonUniformity	glszm	wavelet-HLH
Kurtosis	firstorder	wavelet-LHL

The radscore in the IPA group was 0.964 ± 0.446, whereas that in the PJP group was 1.386 ± 0.524, and the difference between the two groups was statistically significant (P<0.001). ROC curve and AUC were applied to compare the predictive performance between the radscore and machine learning classifiers.

**Figure 4:**
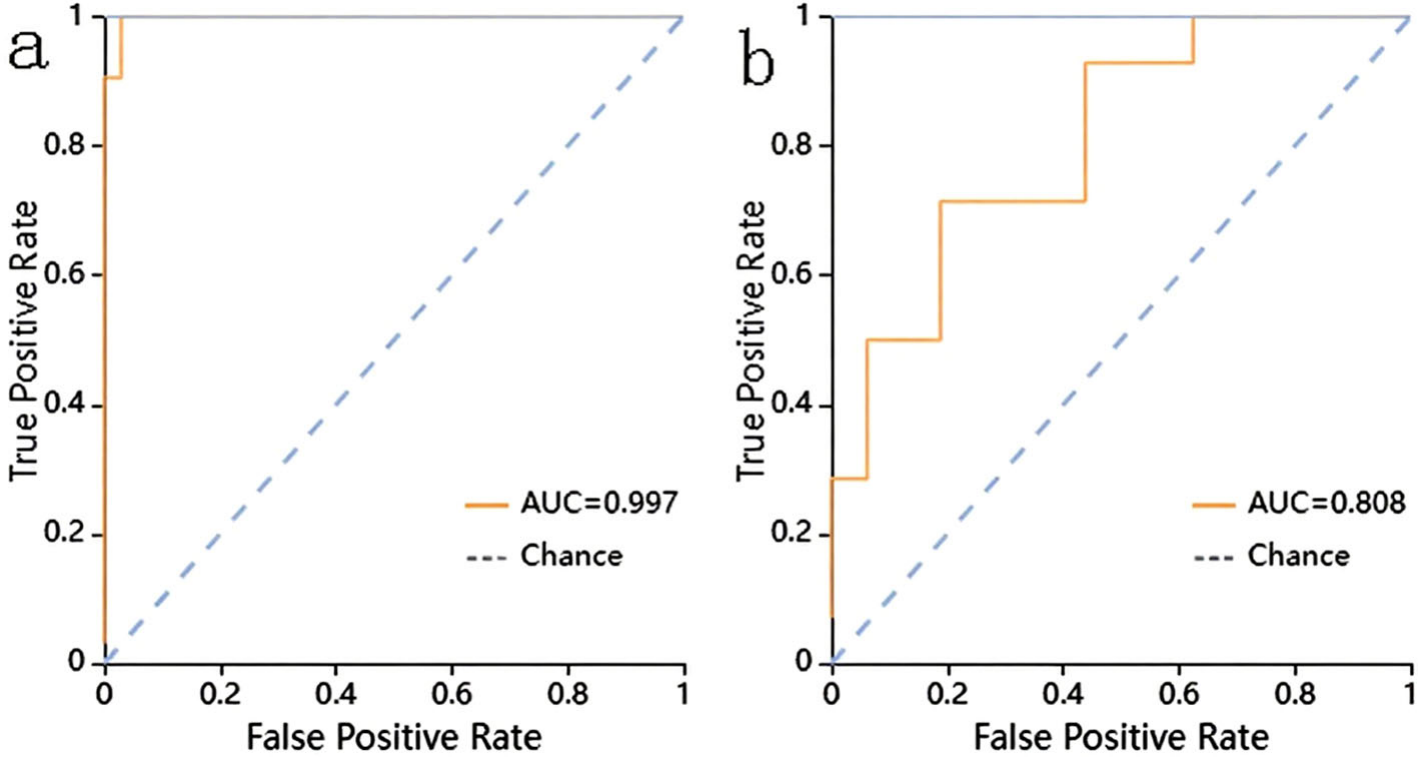
The ROC curve of the XGBoost classifier trained with consolidation area and surrounding GGO area. **(a)** showed the ROC curve in training cohort. **(b)** showed the ROC curve in validation cohort.

**Figure 5:**
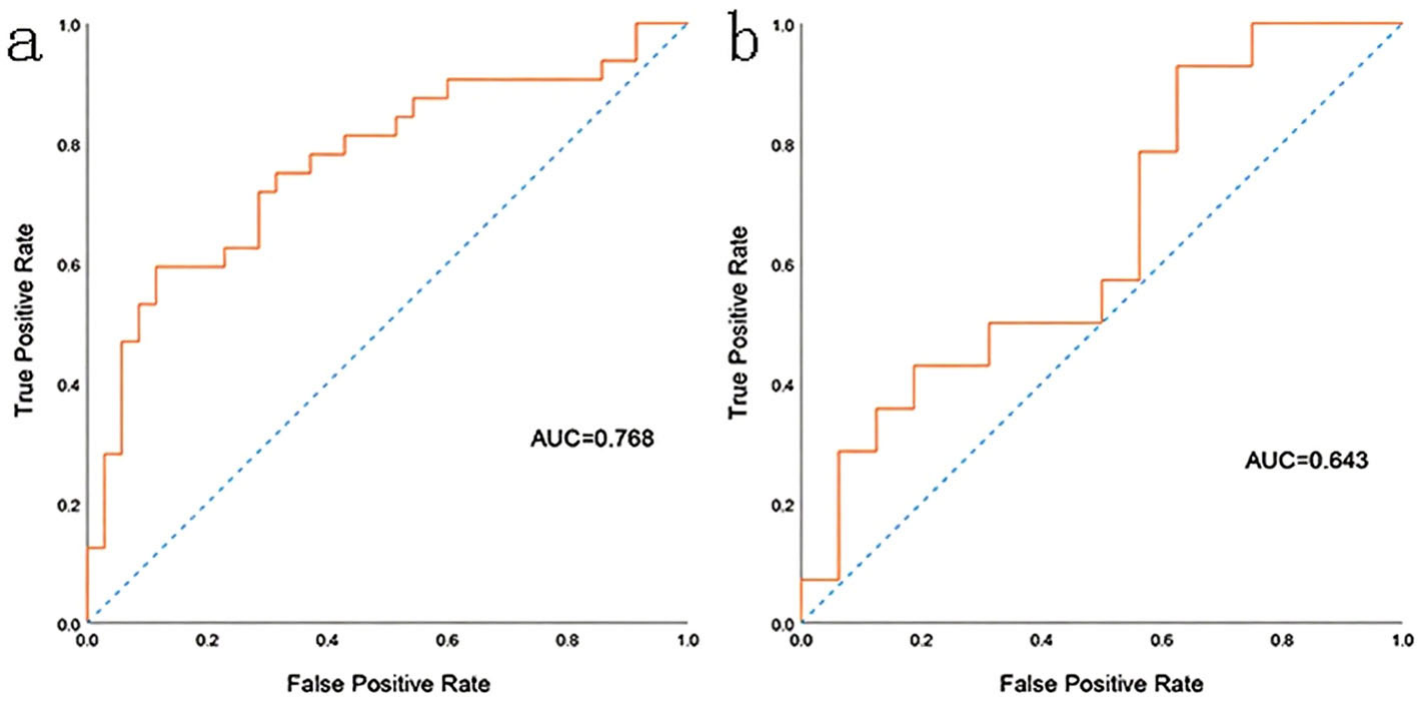
The ROC curve of the radscore model. **(a)** showed the ROC curve in training cohort. **(b)** showed the ROC curve in validation cohort.

The prediction performance using the consolidation+GGO area as the ROI for each classifier in the validation cohort is presented in **Table 3**. The prediction performance of the validation cohort using only the consolidation area as the ROI is presented in **Table 4**. The XGBoost model had an AUC of 0.808 in the validation cohort and performed better than other classifiers trained using the consolidation+GGO area as the ROI and showed better prediction efficacy compared with that of the classifiers trained using only the consolidation area as the ROI. The ROC curve of the validation cohort in the XGBoost classifier using the consolidation+GGO area as the ROI is presented in **Figure 4**. The radscore prediction model had an AUC of 0.643 in the validation cohort (**Figure 5**), indicating that machine learning classifiers had better prediction efficiency than the radscore.

**Table 3:** Prediction performances of classifiers in the validation cohort with the region of interest (ROI) including the consolidation and ground-glass opacity (GGO) regions.

Classifier	AUC	95% CI	Sensitivity	Specificity	Precision	F1-score
KNN	0.790	0.639 - 0.941	0.860	0.630	0.670	0.750
SVM	0.777	0.619 - 0.935	0.790	0.690	0.690	0.730
XGBoost	0.808	0.655 - 0.961	0.710	0.810	0.770	0.740
RF	0.763	0.606 - 0.920	0.790	0.690	0.690	0.730
LR	0.799	0.671 - 0.927	0.930	0.750	0.760	0.840
DT	0.670	0.497 - 0.843	0.710	0.630	0.620	0.670

KNN, K-nearest neighbors; SVM, support vector machine; XGBoost, eXtreme Gradient Boosting; RF, random forest; LR, logistic regression; DT, decision tree.

**Table 4:** Prediction performance of the classifiers in the validation cohort with an ROI consisting of the consolidation region.

Classifier	AUC	95% CI	Sensitivity	Specificity	Precision	F1-score
KNN	0.723	0.548 - 0.898	0.640	0.630	0.600	0.620
SVM	0.790	0.644 - 0.936	0.790	0.810	0.790	0.790
XGBoost	0.661	0.500 - 0.822	0.640	0.810	0.750	0.690
RF	0.681	0.526 - 0.836	0.570	0.880	0.800	0.670
LR	0.790	0.631 - 0.949	0.640	0.810	0.750	0.690
DT	0.567	0.385 - 0.749	0.570	0.560	0.530	0.550

KNN, K-nearest neighbors; SVM, support vector machine; XGBoost, eXtreme Gradient Boosting; RF, random forest; LR, logistic regression; DT, decision tree.

## Discussion

In this study, we used six supervised learning classifiers to establish a radiomics prediction model for differentiating PJP from IPA, and evaluated its predictive performance. The XGBoost classifier trained with the consolidation area and the surrounding GGO area as the ROI had the highest AUC in the validation cohort. This is the first study to demonstrate that such a radiomics model can effectively assist in the differential diagnosis between PJP and IPA.

*P. jirovecii* and *A. fumigatus* are the most important pathogens in fungal pulmonary infections (Fishman and Rubin, 1998). PJP and IPA mainly affect immunocompromised patients, including those with malignant tumors, organ recipients, and patients with human immunodeficiency virus infection. Patients with pulmonary fungal infections often present with non-specific symptoms, such as cough, fever, pleuritic pain, dyspnea, and/or hemoptysis, which affect diagnostic accuracy and may be obfuscated by other bacterial or viral infections (Azoulay et al., 2020). Common diagnostic methods for PJP and IPA include imaging, histopathology, direct microscopic examination of respiratory specimens, galactomannan tests, BDG tests, polymerase chain reaction, and mNGS. However, BDG is a circulating component of various fungal cell walls, and other fungal infections may be confounding factors in BDG assays (Karageorgopoulos et al., 2013). Microscopic examination is highly dependent on sample quality and the response of PJP cysts or trophozoites to staining methods (Fan et al., 2013). This problem persists in the microscopic diagnosis of IPA. Polymerase chain reaction-based diagnostic assays have significantly improved diagnostic efficiency; however, commercial kit prices are relatively high (Zhang F. et al., 2021). The final diagnosis requires the direct detection of pathogens from low respiratory secretions or tissues. BALF is the preferred sample for galactomannan testing in IPA diagnosis (Husain and Camargo, 2019). BALF is also considered to be of the highest quality as a respiratory sample for the diagnosis of PJP and thus has become the current gold standard method of detection (Bateman et al., 2020).

NGS has been successfully applied to detect various infectious pathogens, with a high positive rate for PJP compared with traditional methods. Since mNGS can identify all potential pathogenic microorganisms in BALF samples, we chose NGS results using BALF samples to diagnose and differentiate patients with PJP from those with IPA. However, mNGS has some limitations. It is costly, time-consuming, and affects timely diagnosis and drug treatment.

CT plays a key role in the radiological diagnosis and evaluation of disease activity, response to treatment, and related complications in invasive fungal lung infections (Sanguinetti et al., 2019). Although the CT scan pattern is neither specific nor sensitive for invasive pulmonary fungal infections, it is an effective tool for guiding mycological pre-diagnosis and early initiation of treatment (Bruno et al., 2007). Imaging diagnosis of PJP and IPA remains challenging owing to similar findings in GGO and consolidation areas. Fungal infections should be considered in the differential diagnosis because IPA remains one of the most common infectious mimics of PJP (Cereser et al., 2019). In patients without HIV, PJP shows various findings on CT imaging. Typically, GGO first exists at symptom onset and is symmetrical and predominant in the perihilar and apex regions. Mosaic patterns and architectural distortions are aggravated in cases of ineffective therapy with the same distribution as in the acute phase. GGOs can also exhibit an atypical distribution that predominantly exists in the focal extent or lower lobes (Hardak et al., 2010). Consolidations are common and tend to develop rapidly, typically in patients without HIV, and are occasionally diffuse (Tasaka et al., 2010). Nodules, “crazy paving” patterns, pulmonary cysts, and diffuse alveolar damage are other atypical findings of PJP (Cereser et al., 2019). Similarly, imaging manifestations observed in patients with IPA include GGO, consolidation, nodules, halo signs, and masses (Xu et al., 2012). IPA has vascular infiltrative and airway invasive forms, presenting with different aspects on CT scanning. Typical vascular-invasive CT findings include dense, well-circumscribed lesions >1 cm in size with an early ground-glass attenuation halo (halo sign) (Sanguinetti et al., 2019). The “air crescent sign” is characteristic of a nodular cloudy line permeable crescent that typically appears 1–2 weeks after the onset of IPA (Prasad et al., 2016). Alveolar consolidations or masses, internal low-attenuation or low-density signs, bud patterns, and pleural effusions may also be observed in CT images of the angio-invasive form (Sanguinetti et al., 2019). Airway-infiltrating aspergillosis is characterized by the thickening of the tracheal or bronchial wall and central leaflet nodules that are dendritic, patchy, or predominantly peribronchial consolidation (Franquet et al., 2003). Although the difference between PJP and IPA in CT images is usually non-specific to the human eye, it may present statistically significant radiomic factors.

In our study, radiomics models were effective in differentiating PJP from IPA. Classifiers trained with the ROI comprising both GGO and consolidation areas seem to have better prediction ability than those trained with consolidation areas. The pathophysiological differences between these pathogens may explain the differences in the diagnostic efficacy of the different ROIs. *P. jirovecii* lives almost exclusively in the alveoli, adheres to the surface of the alveolar epithelium of type 1 lung cells, and causes lung damage (Avino et al., 2016). Vascular infiltration in IPA is characterized by hyphae penetrating the vessel wall and the formation of fungal thrombosis, tissue necrosis, and hematogenous dissemination; the halo sign reflects a surrounding hemorrhage of the inflammation site, whereas the air crescent reflects necrosis retraction (Ledoux et al., 2020). Airway-infiltrating aspergillosis is characterized by the destruction of the bronchiolar wall, resulting in the thickening of the trachea or bronchial wall, multiple lobular central nodules, and a bud appearance on CT scans (Franquet et al., 2003).

Radiomics has shown the potential to aid clinical care in disease diagnosis, prognosis prediction, and treatment selection (Gillies et al., 2016). CT-based radiomics is also widely used to detect pneumonia. Gülbay et al (Gülbay et al., 2021) evaluated the discrimination of GGO and consolidation areas between COVID-19 and other atypical pneumonias using radiomic features. They correctly predicted 80% of COVID-19 cases and 81.1% of atypical pneumonia cases. Zhang et al (Zhang M. et al., 2021) constructed a multi-class classification model using radiomics and artificial intelligence for adjunctive empirical antibiotic therapy for bacterial pneumonia in children. In their study, the comprehensive radiomics model using an SVM classifier showed an average AUC of 0.73 in the validation cohort. This is the first innovative study to demonstrate that the radiomics model can effectively differentiate between PJP and IPA. In our study, the radiomics model trained using supervised machine learning classifiers presented good classification efficiency. This may help in early diagnosis, timely initiation of empirical antibiotic use, and personalized precision therapy. This technology has the potential to serve as a foundation for future clinical staging studies, such as assisting in determining disease outcomes through dynamic monitoring of changes in imaging findings. The XGBoost model with the high-precision discrimination framework (AUC=0.808) can provide technical support for subsequent precise classification therapy. The main limitation of this study is the lack of a direct association with clinical staging, which will be emphasized in future work.

In this study, only images of IPA and PJP were collected, the sample size was small, external validation could not be conducted, and other fungal pneumonias were not included in our study; multicenter studies on different kinds of fungal pneumonias should be conducted in the future to take full advantage of radiomics and machine learning. Furthermore, future studies will incorporate clinical severity scoring systems such as APACHE II and SOFA to evaluate the translational potential of our CT-based radiomics model. By correlating the identified discriminative radiomics signatures (e.g., texture heterogeneity in ground-glass opacities or nodule morphology patterns) with dynamic clinical staging scores, we aim to validate their utility in stratifying disease severity and predicting outcomes in immunocompromised hosts with invasive fungal pneumonias.

## Conclusion

The radiomics model shows potential efficacy in differentiating PJP from IPA, which may provide clinicians with early diagnostic evidence for initiating precise antibiotic treatment. The consolidation area with the surrounding GGO area was more suitable for ROI segmentation.

## Data Availability

The original contributions presented in the study are included in the article/supplementary material. Further inquiries can be directed to the corresponding author.
